# Efficacy and Safety of Lomitapide in Homozygous Familial Hypercholesterolaemia: A Systematic Review

**DOI:** 10.31083/j.rcm2305151

**Published:** 2022-04-26

**Authors:** Namin Wei, Yuanhui Hu, Siyu Li, Guoxiu Liu, Nang Zhang, Qiulei Jia, Jingjing Shi, Guozhen Yuan, Huaqiang Zhai

**Affiliations:** ^1^School of Chinese Materia Medica, Beijing University of Chinese Medicine, 102488 Beijing, China; ^2^Department of Cardiovascular Diseases, Guang’anmen Hospital, China Academy of Chinese Medical Sciences, 100053 Beijing, China

**Keywords:** homozygous familial hypercholesterolemia (HoFH), lomitapide, systematic review, efficacy, safety

## Abstract

**Background:**

Homozygous familial hypercholesterolaemia (HoFH) patients have little or no low-density lipoprotein receptor (LDLR) function. HMG-CoA (3-hydroxy-3-methyl glutaryl coenzyme A) reductase inhibitors (statins) and proprotein convertase subtilisin/kexin type 9 (PCSK9) inhibitors have limited lipid-lowering effects, therefore, there is an urgent need to develop new HoFH treatments. In 2012, the US Food and Drug Administration (FDA) approved the administration of lomitapide for lowering low-density lipoprotein cholesterol (LDL-C) levels. However, lomitapide is associated with various gastrointestinal disorders, elevated hepatic alanine aminotransferase (ALT) levels and other adverse reactions, thus, its long-term efficacy and safety in pediatrics and adults should be evaluated. A systematic review conducted in 2017 reported the efficacy and safety of lomitapide in Family hypercholesterolaemia (FH) patients. In this systematic review, we elucidate on the efficacy and safety of lomitapide in HoFH patients.

**Methods:**

A search was conducted in PubMed, Embase, Web of Science and Cochrane library databases to identify valid studies involving lomitapide-treated HoFH patients published before 11th August 2021.

**Results:**

A total of 18 clinical studies involving 120 lomitapide-treated HoFH patients were identified. Lomitapide significantly suppressed LDL-C levels in HoFH patients. Clinical manifestations for lomitapide in children were comparable to those in adults. The most common adverse events were gastrointestinal disturbances and elevated ALT levels. However, most patients tolerated the treatment-associated adverse reactions. Low-fat diets and drug dose adjustments were appropriate measures for controlling the treatment-associated adverse reactions.

**Conclusions:**

In pediatric and adult HoFH patients, lomitapide significantly suppresses LDL-C levels, therefore, it is an important option for HoFH treatment. The most common adverse events of lomitapide treatment include gastrointestinal disorders and elevated hepatic ALT levels. Despite the limitations, lomitapide is feasible for long-term treatment of HoFH patients, with dietary and safety monitoring.

**Registration Number in PROSPERO:**

CRD42021284425.

## 1. Introduction

Familial hypercholesterolaemia (FH), an autosomal dominant disorder of inherited cholesterol metabolism, was systematically described for the first time in 1937 [1, 2]. Homozygous familial hypercholesterolaemia (HoFH) is divided into simple homozygotes (each allele in the same gene carries the same mutation), compound heterozygotes (mutations on each allele in the same gene are different) and double heterozygotes (very rare, mutations on each allele come from different genes) [3, 4, 5].

In a previous study, 20% of patients were found to be administered with a combination of lipid lowering therapy (LLT) and lipid-lowering drugs with 2.7% of the patients had low-density lipoprotein cholesterol (LDL-C) levels below the target value of 1.8 mmol/L [6]. Clinical incidences of HoFH are between 1/160,000 and 1/320,000 [7]. The mechanisms involved in HoFH occurrence are associated with loss-of-function mutations of the two alleles of the low-density lipoprotein receptor (LDLR) gene [8]. Untreated plasma total cholesterol (TC) levels in HoFH patients are usually greater >13 mmol/L. Long-term elevated LDL-C levels are a high-risk factor for atherosclerotic cardiovascular disease (ASCVD) development [9]. The main clinical manifestations of HoFH are premature ASCVD, supralvular aortic stenosis by aortic root atherosclerosis and skin manifestations [10]. Therefore, early, aggressive treatment is important.

Therapeutic options for HoFH include lipid-lowering drugs, lipoprotein plasma exchange, and liver transplantation among other surgical treatments. The mechanisms through which HMG-CoA (3-hydroxy-3-methyl glutaryl coenzyme A) reductase inhibitors (statins) and proprotein convertase subtilisin/kexin type 9 (PCSK9) inhibitors suppress plasma TC levels rely on LDLR [11, 12]. It is difficult for a number of patients with HoFH to achieve the recommended LDL-C level through drug treatment [13].

Lipoprotein apheresis (LA) is the main approach for the treatment of LLT in HoFH patients [14]. However, LDL-C kinetics makes plasma cholesterol levels of patients rebound to baseline levels within 2 weeks [15]. Liver transplantation, which is high risk, is a curative treatment approach for HoFH [16]. Therefore, there is an urgent need for new treatment approaches for HoFH.

The microsomal triglyceride transfer protein (MTP) inhibitor, lomitapide (Juxtapid), which was approved by the US Food and Drug Administration (FDA) in 2012 [17]. However, it does not rely on the expressions of LDLR to reduce LDL-C levels [18]. In addition, the first ANGPTL3 inhibitor, evinacumab, as an adjuvant to other LDL-C reduction therapies for children over 12 years old and adult HoFH patients by FDA in February 2021. MTP is expressed in hepatocytes and intestinal cells where it mediates the triglycerides (TGs) transfer to Apo B particles to form very low-density lipoprotein (VLDL) and chylomicrons. Therefore, inhibition of this reaction reduces VLDL particle foramtion and LDL-C [14]. Experimentally, MTP inhibitors significantly suppressed LDL-C levels in LDLR-deficient Watanabe hereditary hyperlipidemia rabbits (animal models of HoFH) [15, 19]. In adult HoFH patients, lomitapide combined with other LLTs are effective therapeutic options for reducing LDL-C levels, which enabled patients to reach EAS-recommended target levels of LDL-C [20].

Evaluation of the risk profile of lomitapide in clinical trials has confirmed its remarkable efficacy. However, in actual clinical applications and patient management, the benefits and/or risk profiles of lomitapide have not been clearly elucidated. A previous systematic review evaluated the efficacy and safety of lomitapide in hypercholesterolemia. Lomitapide is suitable for improving lipid indices in HoFH patients and severe hypertriglyceridemia recurrent acute pancreatitis [21]. However, the number of included studies and cases in the study was small, while the efficacy and safety of lomitapide in pediatrics were not reported. Moreover, long-term efficacies and safety of lomitapide have not been conclusively determined. Therefore, we elucidate on the long-term efficacy of lomitapide with regards to lipid levels, adverse reactions (gastrointestinal reactions and elevated liver ALT levels) in HoFH patients, as well as on the role of diet in adverse reaction management.

## 2. Methods

### 2.1 Selection Criteria

#### 2.1.1 Inclusion Criteria

The inclusion criteria for studies in this systematic review were: (i) studies whose main objective was assessing oral lomitapidefor HoFH and (ii) clinical cases, case series, retrospective, or prospective studies.

#### 2.1.2 Exclusion Criteria

The exclusion criteria for studies in this systematic were: (i) review-type studies or systematic reviews and (ii) studies whose methodology did not mention positivity to HoFH in study participants.

### 2.2 Search Strategy

Independently, two reviewers (GL and SL) performed the literature search in PubMed, Embase, Web of Science and Cochrane Library databases on 11th August 2021. There were no restrictions on publication dates. In case of disagreements between the two reviewers, a third reviewer (NW) was contacted to make the final decision. Key search words were: (‘lomitapide’ OR ‘Juxtapid’ OR ‘AEGR 733’ OR ‘BMS 201038’) AND (Hypercholesterolemia OR Hypercholesterolemias OR High Cholesterol Levels OR Cholesterol Level, High OR Cholesterol Levels, High OR High Cholesterol Level OR Level, High Cholesterol OR Levels, High Cholesterol OR Elevated Cholesterol OR Cholesterol, Elevated OR Cholesterols, Elevated OR Elevated Cholesterols OR Hypercholesteremia OR Hypercholesteremias). The PubMed search strategy is shown in **Supplementary Table 1**.

### 2.3 Study Selection

For study selection, the titles and abstracts, were filtered to identify the keywords used in the search strategy. Selected studies were placed in a Document Management software (EndNote) to identify duplicate studies. Last, full texts were reviewed to identify studies that met the inclusion criteria. Study selection was independently performed by two reviewers (GL and SL); In case of disagreements between the two reviewers, a third reviewer (NW) was contacted to make the final decision.

### 2.4 Data Extraction

Data on patient characteristics, including age/age range, number of participants and gender, baseline LDL-C levels, HoFH Type, xanthoma, cardiovascular diseases (CVD) events, and disease severity, as well as on lipid-lowering program after lomitapide treatment from the included studies were extracted by two reviewers. Treatment modalities and efficacies of lomitapide therapy, including LDL-C levels before lomitapide administration, LDL-C levels during lomitapide administration, whether it was discontinued, safety, and management of adverse reactions were also recorded.

### 2.5 Risk of Bias

Methodological indices for non-randomized studies (MINORS) tool was used to access the quality of single-arm studies [22]. The Joanna Briggs Institute (JBI) Checklists were used to evaluate the quality of retrospective case series and case reports [23].

### 2.6 Major Outcomes

Efficacy outcomes included changes in LDL-C levels after treatment, compared to baseline levels and lowest LDL-C levels after treatment. Safety outcomes included gastrointestinal symptoms, abnormally elevated liver transaminase levels, and adverse reaction management.

## 3. Results

**Fig. 1.**
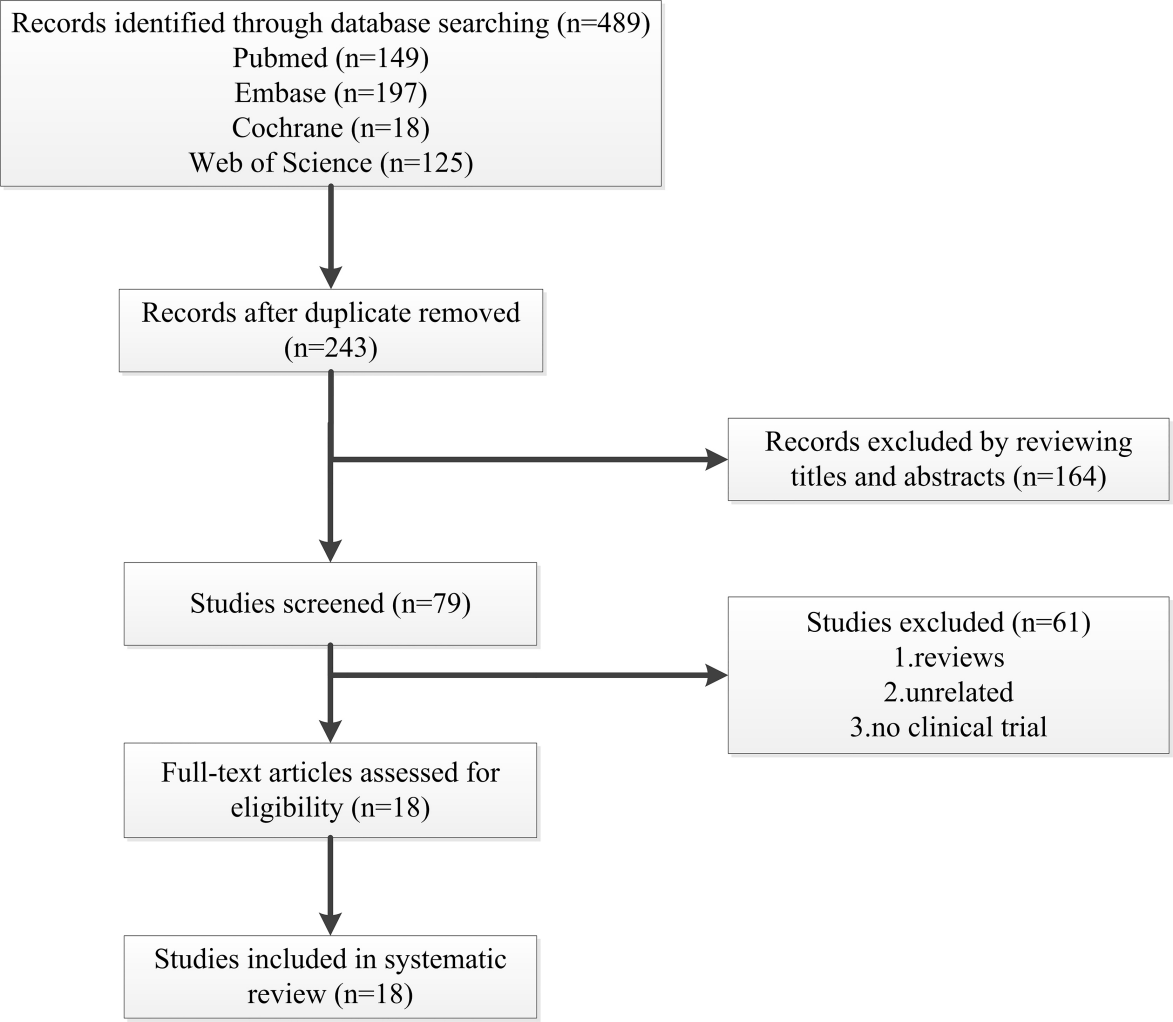
**Flow diagram of studies selected in the present systematic review**.

A total of 489 articles met the initial inclusion criteria. After eliminating duplicates and screening with the exclusion criteria, data analysis was performed on 18 studies with 120 patients. Studies (2 single-arm studies, 2 retrospective case series and 14 case reports) reporting on the changes in lipid levels after lomitapide treatment were selected for analysis. Fifteen studies involving 106 patients reported on adult HoFH [19, 21, 24, 25, 26, 27, 28, 29, 30, 31, 32, 33, 34, 35, 36]. Moreover, 4 studies involved children (14) as study participants [33, 37, 38, 39]. One study included adults and minors as participants [33]. One study did not define the specific age for each patient, and only reported the overall age range: 8–62 years [25]. The study selection processes in this systematic review is presented in Fig. 1 while baseline characteristics of patients are presented in Table 1 (Ref. [19, 21, 24, 25, 26, 27, 28, 29, 30, 31, 32, 33, 34, 35, 36, 37, 38, 39]).

**Table 1.** **Included studies on lomitapide treatment in patients with HoFH**.

Study and study type		Total patients (N)	Patient number	Gender	Age (yr)	Diagnosis	Xanthomas	CVD events
Single-arm studies (2)								
	Harada-Shiba *et al*. [26]	9	1	Female	40	HoFH (4); HeFH (5)	Nm	Nm
			2	Male	46		+	Nm
			3	Male	33		Nm	Nm
			4	Female	52		Nm	Nm
			5	Male	43		+	Nm
			6	Female	35		Nm	Nm
			7	Male	75		Nm	Nm
			8	Female	63		Nm	Nm
			9	Male	66		Nm	Nm
	Cuchel, *et al*. [21]	29		Nm	>18	HoFH	Nm	Nm
Retrospective case series (2)								
	Aljenedil *et al*. [28]	12	1	Male	36	HoFH	Nm	Unstable angina; PTCA; PTCA stent
			2	Male	57	HoFH	Nm	Peripheral vascular disease; left CEA; right CEA; CABG; Aortic valve replacement and aortic root replacement; STEMI aorto-coronary bypass; CABG
			3	Male	22	HeFH	Nm	Aortic valve replacement; Aortic valve prothesis
			4	Female	30	HeFH	Nm	Aortic valve replacement; Aortic valve prothesis
			5	Female	34	HeFH	Nm	Nm
			6	Female	49	HoFH	Nm	CABG
			7	Male	48	HeFH	Nm	CABG; portacaval shunt
			8	Male	36	HoFH	Nm	CAD
			9	Male	39	HoFH	Nm	None
			10	Female	83	HoFH	Nm	None
			11	Female	70	HoFH	Nm	None
			12	Male	29	HeFH	Nm	None
	D’Erasmo *et al*. [27]	15	1	Female	43	ARH	13/15 (+)	CHD (5); aortic valve stenosis (6)
			2	Male	47	ARH		
			3	Female	38	ARH		
			4	Female	48	ARH		
			5	Female	19	HoFH		
			6	Female	23	HoFH		
			7	Female	34	ARH		
			8	Male	29	HoFH		
			9	Female	25	HoFH		
			10	Male	38	HoFH		
			11	Female	67	HoFH		
			12	Male	49	HoFH		
			13	Male	52	HoFH		
			14	Male	32	HoFH		
			15	Female	20	HoFH		
Case reports (14)								
	Ben-Omran *et al*. [37]	11	1	Female	13	HoFH	Nm	Aortic root plaque
			2	Male	12	HoFH	Nm	Left ventricle dilatation; Mild aortic regurgitation and atherosclerotic plaques in both carotid bulbs, and in the common and internal carotid arteries
			3	Male	16	HoFH	+	Nm
			4	Male	7	HeFH	Nm	Aortic plaque
			5	Female	11	HoFH	Nm	Non-critical aortic stenosis/supra-aortic stenosis, and non-obstructive plaques in the carotid arteries
			6	Male	16	HeFH	+	Carotid plaques occluding 25–30% of the carotid lumina
			7	Female	3	HoFH	Nm	Mild aortic thickening; Mild aortic valve regurgitation
			8	Male	14	HeFH	Nm	Mild aortic regurgitation; Bentall procedure
			9	Male	15	HoFH	+	None
			10	Female	8	HoFH	+	Supra-aortic stenosis; Mild tricuspid regurgitation
			11	Male	8	HoFH	Nm	Aortic insufficiency; Focal intimal thickening; thickened tricuspid aortic valve leaflets
	Yahya *et al*. [30]	2	1	Male	25	HeFH	+	Stable moderate aortic valve stenosis; Mild to moderate insufficiency
			2	Female	23	HeFH	–	Stable moderate aortic valve stenosis; Mild insufficiency
	Yahya *et al*. [29]	4	1	Female	29	HoFH	+	The details are not clear
			2	Female	20	HoFH	+	None
			3	Male	36	HoFH	+	The details are not clear
			4	Female	62	HoFH	+	The details are not clear
	Sperlongano *et al*. [31]	2	1	Male	62	HoFH	+	Premature CHD; CABG
			2	Female	52	HoFH	–	AF and atherosclerosis; CHD
	Roeters van Lennep *et al*. [32]	4	1	Female	20	HoFH	Nm	Nm
			2	Female	62	HoFH	Nm	PCI; 4 stents implanted;
			3	Male	42	HeFH	Nm	PCI; Aortic valve replacement
			4	Female	36	HoFH	Nm	2 CABG and mechanical; Aortic valve replacement
	Raper *et al*. [19]	1	1	Female	49	HoFH	+	Premature CAD; AF
	Mahzari and Zarif *et al*. [33]	2	1	Male	17	HoFH	+	CAD
			2	Female	26	HoFH	+	Severe aortic stenosis
	Littmann *et al*. [24]	1	1	Male	26	HoFH	+	Mild to moderate central aortic insufficiency
	Kolovou *et al*. [38]	1	1	Female	8	HoFH	+	Stenotic aortic valve
	Suppressa *et al*. [36]	1	1	Female	28	HoFH	+	ACS; Moderate valvular insufficiency; Intimal thickening and calcified plaques in both carotid arteries
	Cuchel *et al*. [35]	6	1	Female	18	HoFH	Nm	Absent (4); Present (2)
			2	Male	18	HoFH	Nm	
			3	Female	35	HoFH	Nm	
			4	Male	40	HoFH	Nm	
			5	Male	22	HoFH	Nm	
			6	8M/4F	21	HoFH	Nm	
	Kolovou *et al*. [25]	12		Male	8–62	HoFH	+	ASCVD
	Stefanutti *et al*. [34]	7	1	Female	32	HoFH	+	Slight aortic valve disease
			2	Female	24	HoFH	+	CAD+ aortic valve disease; bypass 2009; Aortic and mitral valves replaced 2009
			3	Male	24	HoFH	+	Slight aortic valve disease
			4	Female	25	HoFH	+	Slight aortic valve disease
			5	Female	26	HoFH	+	Moderate aortic valve disease
			6	Female	30	HeFH	+	Slight aortic valve disease
			7	Female	28	HeFH	+	Moderate aortic valve disease
	Chacra *et al*. [39]	1	1	Female	<18	HoFH	+	Atherosclerotic carotid; Aortic valve disease

Nm, Not mentioned; HoFH, Homozygous familial hypercholesterolaemia; HeFH, Heterozygous familial hypercholesterolaemia; ARH, Autosomal recessive hypercholesterolemia; PTCA, Percutaneous transluminal coronary angioplasty; CEA, Carotid endarterectomy; CABG, Coronary artery bypass graft surgery; STEMI, ST-elevation myocardial infarction; CAD, Coronary artery disease; CHD, Coronary heart disease; PCI, Percutaneous coronary intervention; ACS, acute coronary syndrome; AF, atrial fibrillation; ASCVD, Atherosclerotic cardiovascular disease.

### 3.1 Quality Assessment

**Fig. 2.**
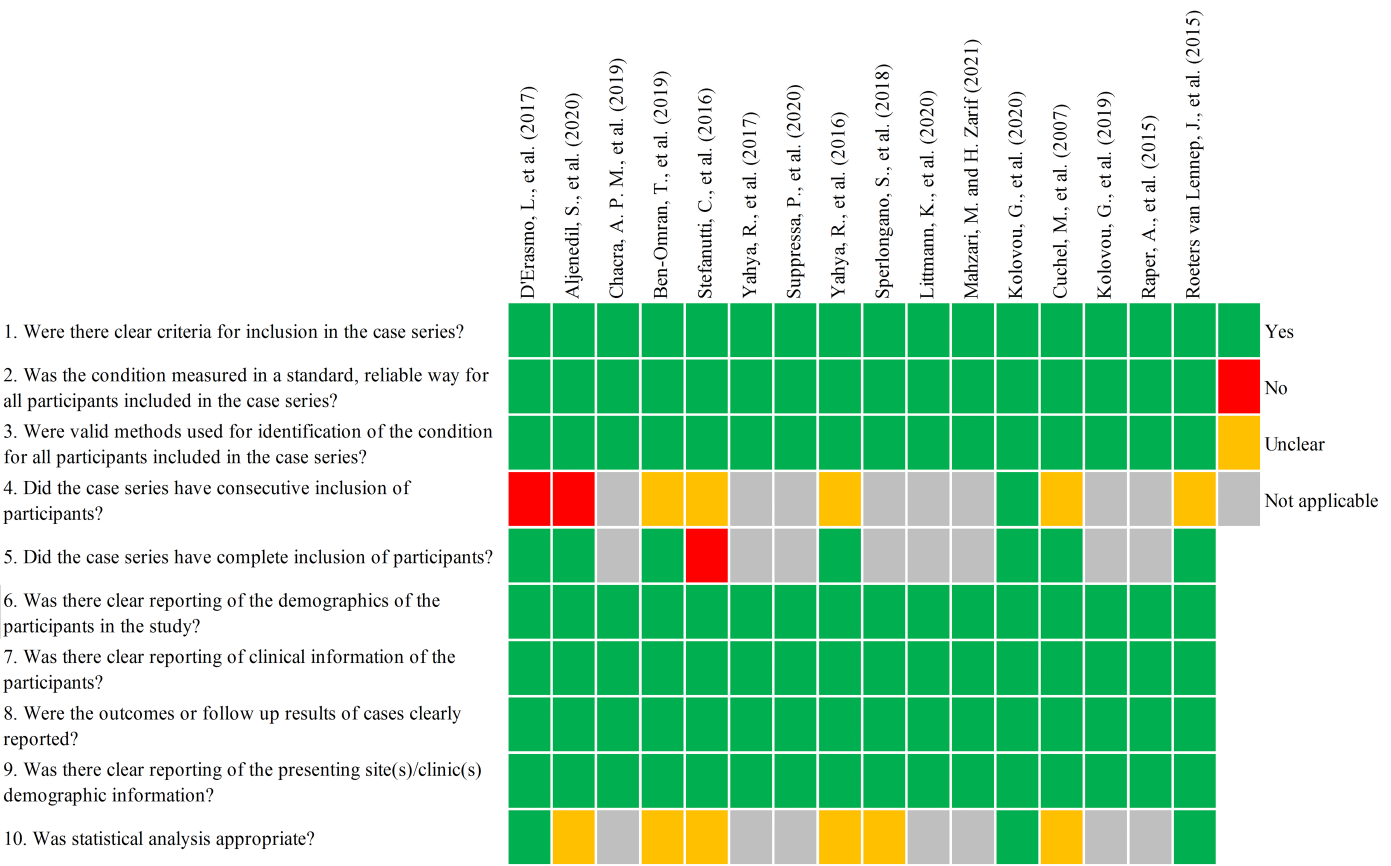
**Individual quality assessment of case series according to the JBI Checklist**. Green, yes; red, no; orange, unclear; grey, not applicable.

**Fig. 3.**
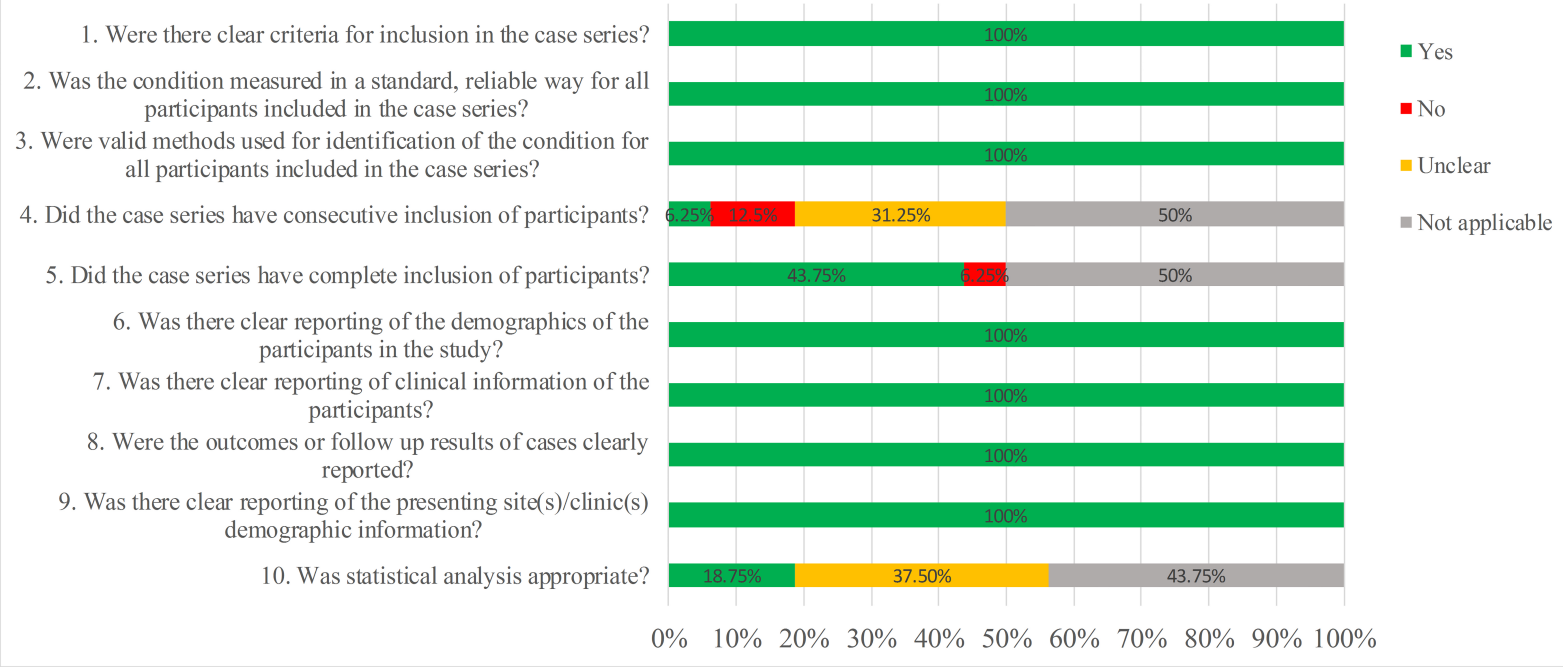
**Overall quality assessment of case series according to the JBI Checklist**.

Quality assessment of the 2 single-arm studies revealed that the first 7 cases were reported and comprehensive in both studies (Table 2, Ref. [21, 26]). Quality assessment of the 2 retrospective case series showed that 7 cases were highly suitable for all the 10 questions (Figs. 2,3).

**Table 2.** **Single-arm studies quality evaluation form**.

	A clearly stated aim	Inclusion of consecutive patients	Prospective data collection	Endpoints appropriate to the aim of the study	Unbiased assessment of study endpoint	Follow-up period appropriate to the study aim	Loss to follow up less than 5%	Prospective calculation of the study size
Harada-Shiba *et al*. [26]	2	2	2	2	2	2	2	0
Cuchel *et al*. [21]	2	2	2	2	2	2	2	0

Items are scored 0 (not reported), 1 (reported but inadequate) or 2 (reported and adequate). The global ideal score non-comparative studies is 16.

### 3.2 Efficacy and Safety Outcomes

Results on the analysis of LDL-C, current LLT regimens, duration of lomitapide treatment, and whether lomitapide treatment was discontinued are presented in Table 3 (Ref. [19, 21, 24, 25, 26, 27, 28, 29, 30, 31, 32, 33, 34, 35, 36, 37, 38, 39]). Lipid-lowering effects of lomitapide on HoFH were explored. CVD complications, lomitapide-related adverse events (AEs) were evaluated as safety outcomes (Table 4, Ref. [19, 21, 24, 25, 26, 27, 28, 29, 30, 31, 32, 33, 34, 35, 36, 37, 38, 39]).

**Table 3.** **Changes in lipid levels before and after lomitapide therapy**.

Study and study type		Patient number	Baseline LDL-C (mmol/L)	Current LLT	Duration of lomitapide treatment	Discountinuation lomitapide treatment (Yes/No)	LDL-C prior to lonitapide	LDL-C at nadir (mmol/L)	LDL-C decrease (%)
Single-arm studies (2)									
	Harada-Shiba *et al*. [26]	1	5.15	rosuvastatin + ezetimibe + LA	56 w	No	5.15	2.98	42%
		2	4.74	rosuvastatin + ezetimibe + colestilan	56 w	No	4.74	4.92	–4%
		3	8.57	ethyl eicosapentaenoic acid + LA	22 w	Yes	8.57	3.57	58%
		4	6.71	ezetimibe + ethyl eicosapentaenoic acid + LA + lomitapide	56 w	No	6.71	0.31	95%
		5	5.18	atorvastatin + ezetimibe + LA + lomitapide	56 w	No	5.18	1.40	73%
		6	5.72	atorvastatin + ezetimibe + probucol + LA + lomitapide	56 w	No	5.72	2.15	62%
		7	3.13	atorvastatin + lomitapide	56 w	No	3.13	1.04	67%
		8	3.47	atorvastatin + ezetimibe + lomitapide	56 w	No	3.47	1.37	61%
		9	3.81	rosuvastatin + ezetimibe + colestilan + LA	56 w	No	3.81	3.39	11%
	Cuchel *et al*. [21]		8.70	statins (27) + ezetimibe (22) + niacin (3) + fibrate (1) + bile acid sequestrant (1) + LA (18) + lompitade (23)	26 w; 56 w; 78 w	7/29 discontinued	8.70	Nm	Nm
Retrospective case series (2)									
	Aljenedil *et al*. [28]	1	9.20	atorvastatin + ezetimibe + LA	37.5 m	Yes	4.90	3.90	58%
		2	18.40	rosuvastatin + ezetimibe + evolocumab + LA	20 m	Yes	8.10	7.30	60%
		3	20.00	rosuvastatin + ezetimibe + LA + lomitapide	25 m	No	5.70	3.80	81%
		4	19.00	atorvastatin + ezetimibe + evolocumab + LA	4 m	Yes	7.30	6.30	67%
		5	10.90	atorvastatin + LA	11.5 m	Yes	11.60	10.70	2%
		6	21.30	atorvastatin + ezetimibe + lomitapide	117 m	No	15.00	4.60	78%
		7	10.60	atorvastatin + ezetimibe + evolocumab + lomitapide	124 m	No	7.10	2.30	78%
		8	10.40	rosuvastatin + ezetimibe + lomitapide	41 m	No	12.30	3.00	71%
		9	13.90	rosuvastatin + ezetimibe + lomitapide	38 m	No	10.20	4.80	65%
		10	18.80	rosuvastatin + ezetimibe + alirocumab + lomitapide	15 m	No	11.40	7.50	60%
		11	11.10	rosuvastatin + ezetimibe + alirocumab + lomitapide	29 m	No	7.50	4.60	59%
		12	10.20	rosuvastatin + ezetimibe + fenofibrate + evolocumab + lomitapide	8 m	No	7.80	5.60	45%
	D’Erasmo *et al*., (2017) [27]	1	12.76*	background therapies + lompitade*	>6 m	No	7.99	3.42*	73%*
		2			>6 m	No	6.06		
		3			>6 m	No	16.06		
		4			>6 m	No	13.16		
		5			>6 m	No	12.17		
		6			>6 m	No	4.35		
		7			>6 m	No	6.92		
		8			>6 m	No	21.83		
		9			>6 m	No	14.27		
		10			>6 m	No	18.80		
		11			>6 m	No	6.27		
		12			>6 m	No	6.89		
		13			>6 m	No	5.49		
		14			>6 m	No	11.89		
		15			>6 m	No	13.36		
Case reports (14)									
	Ben-Omran *et al*. [37]	1	10.85*	atorvastatin + lomitapide	16 m	No	7.74	1.45	Nm
		2		rosuvastatin + ezetimibe + lomitapide + LA	15 m	No	8.44	2.41	Nm
		3		lomitapide + LA	20 m	No	4.84	1.89	Nm
		4		rosuvastatin + lomitapide	15 m	No	21.58	12.07	Nm
		5		atorvastatin + ezetimibe + lomitapide	48 m	No	11.47	5.98	Nm
		6		rosuvastatin + ezetimibe + lompitade	15 m	No	6.29	0.60	Nm
		7		atorvastatin + ezetimibe + lomitapide	12 m	No	16.81	6.11	Nm
		8		atorvastatin + ezetimibe + lomitapide	22 m	No	5.78	1.94	Nm
		9		atorvastatin + ezetimibe + lomitapide + LA	18 m	No	2.1	1.61	Nm
		10		atorvastatin + ezetimibe + lomitapide	19 m	No	16.32	11.42	Nm
		11		atorvastatin + ezetimibe + lomitapide	19 m	No	18.26	11.91	Nm
	Yahya *et al*., (2017) [30]	1	19.60	atorvastatin + ezetimibe + lomitapide	5 y	No	9.00	1.71	91%
		2	17.80	atorvastatin + lomitapide	3 y	No	8.80	0.75	96%
	Yahya *et al*., (2016) [29]	1	Nm	atorvastatin + ezetimibe + lomitapide	9.5 w	Yes	14.50	2.40	Nm
		2	Nm	atorvastatin + cholestagel + lomitapide	36.5 w	No	14.10	0.77	Nm
		3	Nm	simvastatin + ezetimibe + lomitapide	9 w	No	3.90	4.50	Nm
		4	Nm	questran + modalim +lomitapide	9 w	Yes	12.90	2.00	Nm
	Sperlongano *et al*. [31]	1	7.64	background therapies + lompitade	52 w	No	7.64	Nm	Nm
		2	5.49	rosuvastatin + ezetimibe + LA + lomitapide	55 w	No	2.77	Nm	Nm
	Roeters van Lennep* et al*. [32]	1	14.11	atorvastatin + lomitapide	50 w	No	14.11	2.40	83%
		2	10.35	lomitapide (stopped permanently)	44 w	Yes	10.35	0.77	93%
		3	7.16	lomitapide + rosuvastatin + ezetimibe + colesevelam	20 w	No	7.16	4.50	37%
		4	1.30	LA + simvastatin + ezetimibe + Lomitapide	24 w	No	7.30	2.00	–54%
	Raper *et al*. [19]	1	5.78	lomitapide + rosuvastatin + ezetimibe	>5 y	No	16.50	0.73	87%
	Mahzari. And Zarif *et al*. [33]	1	16.50	Lomitapide (stopped) + rosuvastatin + ezetimibe + evolocumab	>1 y	Yes	13.3	2.20	87%
		2	15.30	rosuvastatin + ezetimibe + lomitapide (the patient died)	3 m–1 y	No	15.30	6.90	55%
	Littmann *et al*. [24]	1	18.50	lomitapide + LA	>1 y	No	3–4	Nm	Nm
	Kolovou *et al*. [38]	1	26.00	lomitapide + rosuvastatin + ezetimibe + colesevelam	2 y	No	26.00	10.00	62%
	Suppressa *et al*. [36]	1	7.77	rosuvastatin + ezetimibe + lomitapide	2 y	No	14.04	1.17	85%
	Cuchel *et al*. [35]	1	12.43	Nm	16 w	No	Nm	5.80	53%
		2	20.44	Nm	16 w	No	Nm	9.92	51%
		3	15.77	Nm	16 w	No	Nm	10.44	34%
		4	16.50	Nm	16 w	No	Nm	7.80	53%
		5	13.83	Nm	16 w	No	Nm	5.21	62%
		6	16.47	Nm	16 w	No	Nm	7.93	52%
	Kolovou* et al*. [25]		23.31*	LL drugs + lomitapide + LA (9/12)	3–24 m*	2/12 stopped*	7.46*	1.81*	92%
	Stefanutti *et al*. [34]	1	Nm	LA + lomitapide	Nm	No	Nm	1.27	Nm
		2	Nm	LA + lomitapide	Nm	No	Nm	1.92	Nm
		3	Nm	Lomitapide + LA	Nm	No	Nm	3.89	Nm
		4	Nm	LA + lomitapide	Nm	No	Nm	3.89	Nm
		5	Nm	LA + atorvastatin + ezetimibe + lomitapide	Nm	No	Nm	2.62	Nm
		6	Nm	lomitapide + LA	Nm	No	Nm	1.61	Nm
		7	Nm	lomitapide + LA	Nm	No	Nm	3.26	Nm
	Chacra* et al*. [39]	1	26.13	atorvastatin + ezetimibe + lomitapide	49 m	No	11.09	5.98	77%

Nm, Not mentioned; *, Represents the level or protocol of the study; LLT, Lipid lowering therapy; LDL-C, Low-Density Lipoprotein Cholesterol; LA, Lipoprotein apheresis; w, Week; m, Month; y, Year; LDL-C decrease (%) = (Baseline LDL-C – LDL-C at nadir)/Baseline LDL-C × 100%.

**Table 4.** **Lomitapide-associated adverse events and their management**.

Study and study type		Total patients (N)	Patient number	AEs	Notes on AEs management
Single-arm studies (2)					
	Harada-Shiba *et al*. [26]	9		GIs (8); Increased hepatic enzymes (3)	Reducing the dose or discontinuation of lomitapide treatment
	Cuchel *et al*. [21]	29		GIs (27); Increased hepatic enzymes (4); ACS and AP and LRTI (1); Elective hysterectomy for menorrhagia (1); Chest pain (1)	Reducing the dose or temporary interruption of treatment
Retrospective case series (2)					
	Aljenedil *et al*. [28]	12	1	Increased hepatic enzymes	Reducing the dose then discontinuation
			2	Noncompliance; GIs	Discontinuation
			3	Diarrhea	Dose adjustment
			4	Moderate diarrhea	Discontinuation
			5	Noncompliance; Moderate diarrhea	Discontinuation
			6	Increased hepatic enzymes; Moderate nausea and diarrhea	Dose adjustment
			7	Moderate diarrhea and nausea	Dose adjustment
			8	None	None
			9	Moderate diarrhea only upon early	No drug adjustment
			10	Moderate vomiting and diarrhea	Dose adjustment then discontinuation
			11	Moderate nausea and diarrhea	None
			12	Tired 3 days after starting lomitapide; normalized after; Rare abdominal discomfort	None
	D’Erasmo *et al*. [27]	15		GIs	Dietary modifications; Dose adjustment; Antidiarrheic medications
Case reports (14)					
	Ben-Omran *et al*. [37]	11	1	Mild GIs	Can tolerate
			2	Nm	Nm
			3	Mild GIs	Can tolerate
			4	None	None
			5	Mild GIs	Dietary modifications; Adjusted dosage
			6	Mild GIs; Increased hepatic enzymes	Adjusted dosage
			7	None	None
			8	Increased hepatic enzymes	Adjusted dosage
			9	None	None
			10	None	None
			11	None	None
	Yahya *et al*. [30]	2	1	None	None
			2	None	None
	Yahya *et al*. [29]	4	1	GIs	Dietary modifications
			2	GIs	Dietary modifications
			3	GIs	Dietary modifications
			4	GIs; Increased hepatic enzymes	Dietary modifications; Stopped
	Sperlongano *et al*. [31]	2	1	Mild GIs	Dietary modifications; Antidiarrheic medications
			2	Mild GIs	Dietary modifications; Adjust the dosage
	Roeters van Lennep *et al*. [32]	4	1	Mild GIs	Dietary modifications
			2	Mild GIs; Increased hepatic enzymes	Antidiarrheic medications; Stopped permanently
			3	None	None
			4	None	None
	Raper *et al*. [19]	1	1	Mild GIs; Increased hepatic enzymes	Adjusted dosage
	Mahzari. and Zarif *et al*. [33]	2	1	Nm	Nm
			2	Nm	Nm
	Littmann *et al*. [24]	1	1	GIs	Adjusted dosage
	Kolovou *et al*. [38]	1	1	None	None
	Suppressa *et al*. [36]	1	1	GIs	Adjusted dosage
	Cuchel *et al*. [35]	6		GIs; Increased hepatic enzymes	Dietary modifications; Adjusted dosage
	Kolovou *et al*. [25]	12		GIs; Increased hepatic enzymes	Nm
	Stefanutti *et al*. [34]	7	1	Mild GIs	Dietary modifications
			2	Increased hepatic enzymes	Temporary interruption; Diet modification
			3	None of note	None of note
			4	None of note	None of note
			5	None of note	None
			6	None of note	None
			7	None of note	Diet modification
	Chacra *et al*. [39]	1	1	GIs	Diet modification; Adjusted dosage

Nm, Not mentioned; AEs, Adverse events; GIs, Gastrointestinal symptoms; ACS, Acute coronary syndrome; AP, Atherosclerotic plaque; LRTI, lower respiratory tract infection.

#### 3.2.1 Adult HoFH Patients

Lipid-lowering efficacies of lomitapide combined with other LLTs in adult HoFH patients were investigated. Cuchel *et al*. [21] assessed the efficacy and safety of lomitapide in a single-arm study comprising 29 adult HoFH patients. Among the 29 patients, 23 completed the efficacy period (26 weeks) and the full study (78 weeks). The main treatment involved increasing lomitapide dose based on efficacy of the original LLT. Gradually, the lomitapide dose was increased from 5 mg/d to 60 mg/d. Mean LDL-C levels ranged from 8.7 mmol/L at baseline to 4.3 mmol/L at week 26. Eight patients had LDL-C levels below 2.6 mmol/L. At weeks 56 and 78, the decrease in LDL-C levels were 44% and 38% respectively. Gastrointestinal symptoms, which were effectively alleviated by dietary adjustments or dose reductions. Were the most common AEs. ALT activities ≥3 × upper limit of normal value (ULN) was reported in 10 of 29 patients. Liver functions were not affected by temporary drug withdrawals or dose reductions. None of the patients permanently withdrew treatment due to abnormal liver functions. In a single-arm study of performed by Harada-Shiba *et al*. [26], initially, lomitapide was administered at a dose of 5 mg/d, after which it increased to 60 mg/d within 14 weeks. Nine patients underwent the efficacy phase, with eight of these patients completing the 56 weeks period. At week 26, mean LDL-C levels had decreased by 42%, from a baseline level of 199 mg/dL to 118 mg/dL. Moreover, at week 26, LDL-C levels were 50% low in 5 of the 9 patients. Relative to baseline levels, LDL-C levels at week 56 were 38% low. These findings imply that lomitapide combined with other LLTs significantly suppressed LDL-C and Apo B levels in Japanese adult HoFH patients. Two patients exhibited xanthoma alleviation after 56 weeks. ALT levels were ≥3 × ULN in 3 of the 9 patients, with normal ALT levels being restored in two patients through dose reductions. Most cases (5/9) had liver fat levels below 10%.

D’Erasmo *et al*. [27] conducted two retrospective studies and obtained clinical as well as biochemical data from 15 HoFH patients treated with LLT and lomitapide. During treatment, average LDL-C levels were 426 ± 204 mg/dL. At the last visit, 60% of the patients had LDL-C levels <100 mg/dL while 46.6% had LDL-C levels <70 mg/dL. Due to marked reductions in LDL-C levels, 80% of the patients who had received LA stopped receiving it at follow-up. A wide range (13–95%) of LDL-C level reduction was noted, which may be genotype-associated. During follow-up, about 53.3% of the patients reported at least one episode of diarrhea, ALT levels ≥5 × ULN or treatment discontinuation due to severe side effects. Several patients were followed up for more than 1 year. In the study, six patients were treated for more than two years. Aljenedil *et al*. [28] reported that the twelve HoFH patients in the study had an average age of 44 ± 18 years. All the twelve patients were treated with statins and ezetimibe, while 5 patients were treated with LA. Lomitapide suppressed LDL-C levels by 38%, but was discontinued in three patients due to intolerable gastrointestinal side effects. After dose reduction, adverse events were alleviated in two patients whose ALT levels ≥3 × ULN.

Littmann *et al*. [24] reported a case of a female patient diagnosed with HoFH at the age of 6 and treated with lomitapide. The patient completely lacked normal LDLR activities and did not exhibit any responses to statin therapy. At 7 years of age, the patient was treated with LA. When lomitapide was combined with LA treatment, LDL-C levels significantly improved. Then, the LA dose was reduced from 2 times a week to once every 2 weeks, after which the quality of life of the patient improved. The patient did not present with any AEs. Raper *et al*. [19] conducted a case study of a 49-year-old woman with HoFH and a complex cardiovascular history who was treated with lomitapide for 5 years in combination with other LLTs. Long-term lomitapide administration significantly suppressed LDL-C levels to <70 mg/dL. Due to significant reductions in LDL-C levels, LA and colesevelam were discontinued. Therefore, lomitapide (60 mg/d) + rosuvastatin (40 mg/d) + ezetimibe (10 mg/d) were used as the main LLT regimen. After treatment, the patient’s quality of life improved. The patient tolerated lomitapide, with a few side effects observed during treatment. In 2020, Kolovou *et al*. [25] investigated the characteristics of 12 HoFH patients. After treatment, LDL-C levels further reduced by 56.8%, compared to patients treated with lipid-lowering drugs alone. Moreover, compared to levels in patients with a combination of lipid-lowering drug and LA, there was a 54% decrease in LDL-C levels. In this study, HoFH patients treated with the maximum tolerable dose of lipid-lowering agents and LA and who did not achieve normal LDL-C levels were subjected to a combination therapy of lomitapide to decrease LDL-C levels. In 2016, Yahya *et al*. [29] conducted a study involving on 4 HoFH patients treated with increasing lomitapide doses. They found that lomitapide reduced LDL-C levels (range –34– –89%), and all patients presented with gastrointestinal symptoms during treatment. These side effects were alleviated via the intakes of low-fat diets. Due to non-adherence to treatment, lomitapide treatment of patient 1 was discontinued. During treatment, patient 4 exhibited ALT levels ≥5 × ULN, which were restored to normal levels after lomitapide discontinuation. Moreover, a study involving patients with two compound HeFH diagnosed at childhood and treated using LLT [37] revealed that after lomitapide administration at a dose of 20 mg/d, LDL-C levels for patient 1 decreased by 45%. Patient 2 showed an 87% maximum reduction in LDL-C levels after 30 mg/d lomitapide administration. Although significant reductions in LDL-C levels were achieved at an early age in both patients, LDL-C levels were still above 2.6 mmol/L.

Sperlongano *et al*. [31] reported findings on two lomitapide-treated HoFH patients. Compared to baseline levels, after lomitapide administration, there was a 78% reduction in LDL-C levels in patient 1 and an 86% reduction in patient 2. LA therapy was stopped in patient 2. During lomitapide administration, two patients presented with mild gastrointestinal symptoms. Side effects were alleviated by a low-fat diet and antidiarrheal medications. Patients did not show any elevations in ALT and liver fat levels. These findings indicate that lomitapide administration to patients in middle and early stages can reduce LDL-C levels and the risk of CVD. Roeters* et al*. [32] performed a study involving 4 adult HoFH patients. Each patient was administered with lomitapide and subjected to routine follow-up. In all 4 patients, LDL-C levels were reduced by 35 to 73%, with 3 of the 4 patients presenting with gastrointestinal AEs that were alleviated via appropriate dieting. During the whole study period, three patients were administered with lomitapide, while lomitapide administration was stopped in one patient due to elevated ALT levels, which were restored to normal levels after treatment withdrawal. Mahzari *et al*. [33] conducted a case study involving two HoFH patients treated with lomitapide in Saudi Arabia. After lomitapide treatment, LDL-C levels of patient 2 decreased from 15.3 mmol/L to 6.9 mmol/L. After one year of lomitapide administration, patient 2 died, which was attributed to cardiovascular surgery associated complications. However, the two patients did not show severe lomitapide-associated side effects. Suppressa *et al*. [36] reported a case of a 28-year-old female HoFH patient who had been diagnosed with xanthoma at age 2. The patient rejected LA therapy, therefore, LLT treatment was initiated using statins, ezetimibe and evolocumab, however, this therapy did not significantly decrease LDL-C levels. Treatment with increasing lomitapide doses (up to 30 mg/d) was initiated at month 24 of follow-up, resulting in decreased LDL-C level to 45 mg/dL. During lompitade treatment, the patient did not present CVD complications.

Cuchel *et al*. [35] conducted study in which six HoFH patients aged 18–40 years were treated with increasing lomitapide doses (0.03, 0.1, 0.3, 1.0 mg/kg/d). Four weeks prior to lompitade treatment, LLT therapy was suspended for 4 weeks in each group. After a 4-week drug elution period, patients returned for a final follow-up. All patients tolerated lomitapide treatment to a maximum dose of 1.0 mg/kg/d, which reduced LDL-C levels by 50.9% and Apo B levels by 55.6%, compared to baseline levels. The most severe AEs included elevated ALT levels and hepatic fat accumulation. Stefanutti *et al*. [34] reported on the effects of administration of lomitapide in addition to LA in 7 adult HoFH patients. In most (5/7) patients, the dose range of lomitapide was 10–30 mg/d. One patient received 60 mg/d lomitapide whereas another patient received 5 mg/d lomitapide. LDL-C levels reduced by more than 50% in 3 patients. Six patients receiving LA in this trial showed a reduction in dosing frequency, with three patients permanently discontinuing LA intake. Notably, patients who received the lowest lomitapide dose of did not achieve significant benefits from treatment. Gastrointestinal AEs were managed by a low-fat diet.

#### 3.2.2 Paediatric HoFH Patients

One study investigated the efficacy and safety of lomitapide in paediatric HoFH patients. Ben-omran *et al*. [37] reported on lomitapide outcomes in paediatric HoFH patients for the first time. The mean age for patients in the study was 11.6 ± 1.1 years. About 64% of patients were male and they exhibited ASCVD signs. The mean lomitapide dose administered to this cohort was 24.5 ± 4.3 mg/d while the mean exposure time was 20.0 ± 2.9 months in addition to the original lipid-lowering regimen. The LDL-C levels were markedly reduced to a minimum of 176.7 ± 46.3 mg/dL (mean baseline: 419.0 ± 74.6 mg/dL). After lomitapide treatment, six patients presented the recommended target level for paediatric patients below 135 mg/dL, including five patients with reduced LA dosage frequency. Severe AEs were gastrointestinal reactions. Three patients showed deviations in liver function tests, which could not be alleviated with intervention. Patients and carers were advised that lomitapide should be accompanied with a low-fat diet whereby less than 20% of total daily energy is derived from fat. Mahzari *et al*. [33] reported on two HoFH patients in Saudi Arabia who had been treated with lomitapide. Patient 1 was a juvenile, and lomitapide administration significantly decreased LDL-C levels (87%) from 16.5 mmol/L to 2.2 mmol/L. Kolovou *et al*. [38] reported a case of an 8-year-old HoFH boy with large tuberous xanthoma of the hand, elbow, hip, knee, and foot. Lomitapide was added to the conventional LLT therapy at a dose of 40 mg/d (steadily increasing from 2.5 mg/d). After 2 years of treatment, the thickness, hardness, size and colour intensity of xanthoma was reduced by 50%. During lomitapide administration, the patient did not present any side effects. Chacra* et al*. [39] reported a case of a 7.6-year-old female HoFH patient who received lomitapide for 49 months. Lomitapide was added to a basal therapy comprising ezetimibe and atorvastatin. At an average dose of 20 mg/d, lomitapide reduced LDL-C levels by 37% in this patient. Growth and development for children were normal; however, the progression of subclinical carotid atherosclerosis or aortic valve disease was observed. The drug was well tolerated by the patient, who presented with diarrhea after 30 mg/d lomitapide administration. The diarrhea was alleviated when the dose was titrated down to 20 mg/d. In addition, the patient was fed on a low-fat diet, and liver enzyme changes as well as liver steatosis did not occur. The patient was oriented to follow a restricted fat diet (20% of calories, to prevent steatorrhea) and then to start supplementation of fat-soluble vitamins and essential fatty acids.

## 4. Discussion

Eighteen clinical trials involving 120 lomitapide-treated HoFH patients were included in this study (2 single-arm studies, 2 retrospective case series and 14 case reports). Lomitapide significantly reduced LDL-C levels in HoFH paediatrics and adults, but also increased the risk for gastrointestinal reactions, ALT elevations, and liver fat accumulation. However, the adverse effects were controllable.

Lomitapide can significantly reduce LDL-C levels and the risk of CVD during HoFH treatment [40]. Modeling data in adult patients revealed that early interventions with lomitapide has the potential to increase the life expectancy and delay the onset of the first major adverse cardiovascular events [41]. The potential of lomitapide in long-term HoFH management has been evaluated. Among the included studies in this systematic review, Raper *et al*. [19] reported on lomitapide administration for >5 years, implying that lomitapide is feasible for long-term HoFH treatment with careful attention to diet and safety monitoring of patients. D’Erasmo *et al*. [27] reported that many patients were followed up for more than 1 year, and 6 of them were treated for more than 2 years. Kolovou *et al*. [38] reported that, an 8-year-old patient did not experience any side effects after lomitapide administration for 2 years. Chacra *et al*. [39] reported a 37% reduction in LDL-C levels in patients treated with lomitapide for 49 months, with no uncontrolled adverse reactions. Lomitapide is also an effective cholesterol lowering agent with a good safety profile.

Compared to previous systematic reviews, this study informs on the efficacy and safety of lomitapide in paediatrics with HoFH. Early identification of CVD children and their timely referral to specialists are crucial active LLT measures for reducing CVD risks [3]. Currently, lomitapide is not permitted for use in children, however, clinical studies have been conducted through expanded access programs or on a designated patient basis. Clinical trials involving HoFH patients treated with lomitapide included in this study showed that lomitapide significantly reduces LDL-C levels in HoFH patients, reduces the frequency of LA treatment, reduces the risk of early CVD, and improves the quality of life for patients. There were few lomitapide-associated adverse reactions, with gastrointestinal disorders being the most important. However, adverse reactions could be controlled using low fat diets or through treatment dose adjustment.

Ben-omran *et al*. [37] reported that lomitapide has a good efficacy in pediatric HoFH patients, with 6 of 11 patients achieving the recommended target of 135 mg/dL. The frequency of LA was decreased in 5 patients, whereas clinical manifestations of the drug were like those in adult patients. Yahya *et al*. [30] reported that 2 HoFH patients diagnosed at a young age and administered with LLT (including lomitapide) were without AEs and CVD had not yet occurred on the patients. Mahzari *et al*. [33] documented that one HoFH patient in Saudi Arabia, who had received lomitapide, showed an 87% reduction in LDL-C levels without AEs. Kolovou* et al*. [38] documented that the xanthoma of an 8-year-old boy with HoFH was improved after lomitapide administration, without any side effects. The first reported long-term (49 months) use of lomitapide in children with HoFH was by Chacra *et al*. [39]. Lomitapide reduced LDL-C levels by 37%, however, diarrhea occurred during lomitapide use, whereas alterations in liver enzyme levels and hepatic steatosis were controlled through dose reductions and a low-fat diet.

A combination therapy involving statins, ezetimibe and LA is the most effective LLT therapy for HoFH patients [5]. In HoFH patients, statin monotherapy does not significantly reduce LDL-C levels; however, it reduces LDL-C levels by an average of 26% and, significantly reduces CVD events as well as all-cause mortality [12]. Treatment of HoFH with LLTs does not reduce LDL-C levels to required levels, therefore, due to these limitations, LA is the standard treatment option for HoFH. J. Višek *et al*. [42] analyzed data on FH patients treated with LA for 15 years. They found that long-term LA treatment improved lipid levels and endothelial dysfunction, without cardiovascular complications. However, LA treatment is expensive and requires a long treatment period as well as high patient compliance [43]. Moreover, due to the frequency of treatment (at least two weeks) and the need to maintain vascular access, not all patients are eligible for monotherapy. This technology requires highly specialized facilities and is not available in most countries [44]. LA treatment may also present technical, clinical, and social challenges, especially in children [45, 46]. Although the current lipid-lowering drugs and LA can significantly improve the prognostic outcomes for HoFH patients, they result in LDL-C levels above target levels in most patients [12]. Therefore, new drugs are urgently needed for HoFH treatment. Lomitapide is characterized by a high efficacy and tolerability, therefore, it is an alternative to LA for several patients awaiting liver transplantation. Littmann *et al*. [24] reported that since it is associated with significantly improved LDL-C levels and it markedly reduces the frequency of LA administration, lomitapide can be used as the drug of choice for HoFH. Stefanutti [34] reported that in addition to LA treatment,7 adult HoFH patients were treated with lomitapide, resulting in reduced LA treatment frequencies in 6 patients and permanent withdrawal of LA in 3 patients. This indicates that lomitapide can be used as an adjunct treatment to LA in HoFH.

Lomitapide-associated adverse reactions may lower patient adherence to treatment and limit the use of the maximum tolerable dose, potentially reducing its efficacy. Adverse reactions included gastrointestinal symptoms, elevated hepatic ALT levels, and accumulation of liver fat, which may lead to steatohepatitis or liver fibrosis [47]. Cuchel *et al*. reported on AEs in at least 90% of patients treated with lomitapide, with gastrointestinal symptoms (diarrhea, nausea, vomiting, or indigestion) being the most common [16, 21, 35, 48].

Most of the lomitapide-associated adverse reactions can be alleviated through different management approaches. For instance, gastrointestinal symptoms can be minimized through intakes of low-fat diets (20% of energy is obtained from fat). Clinical use of lomitapide can be regulated by gradually increasing the dose under tolerable levels or decreasing the dose when necessary [49]. The effects of lomitapide precursors in healthy volunteers (n = 48) have been investigated. It revealed that gastrointestinal AEs were significantly associated with high-fat diets [50]. Due to associated adverse reactions, lomitapide prescription requires intense patient education and liver function monitoring during treatment [5, 51].

## 5. Limitations

This study is associated with some limitations. First, some unpublished studies were not included in the search, which may result in publication bias. Second, lomitapide is an orphan drug used for the treatment of orphan diseases. Studies on lomitapide as an adjunct to other LLTs are mainly small clinical sample size studies. Currently, due to ethical limitations, there are no long-term large randomized clinical trials on efficacies of lomitapide on hard clinical endpoints in HoFH. Therefore, assessment of the safety of lomitapide is limited. Third, the information in some studies were incomplete. Some studies did not report on differences in exposure time of lomitapide treatment. Some trials did not report the data on dietary fat intake by patients [30]. In addition, no large-scale data are available on lomitapide use in HoFH children. Only 4 case reports documented on the use of lomitapide in infant patients or in HoFH children [33, 37, 38, 39]. Therefore, the efficacy and safety of lomitapide in children and infants with HoFH should be explored further.

## 6. Conclusions

Lomitapide is an effective treatment option for significantly reducing LDL-C levels in adult patients with HoFH; however, further data are needed in children. Moreover, lomitapide is suitable for long-term use as an adjunct therapy for patients treated with LA to reduce the frequency of LA dosage. If HoFH patients treated with the maximum tolerable dose of lipid-lowering drugs and LA do not achieve normal LDL-C levels, lomitapide can be administered as an adjunct drug if HoFH patients treated with the maximum tolerable dose of lipid-lowering drugs and LA do not achieve normal LDL-C levels. Lomitapide is associated with adverse reactions, mainly manifested as gastrointestinal reactions, such as diarrhea, nausea, and vomiting. In addition, elevated ALT levels in the liver are associated with high levels of lomitapide administration. However, adverse reactions were alleviated through diet management, regular monitoring, and dosage adjustment.

## Supplementary Material

Supplementary material associated with this article can be found, in the online version, at https://doi.org/10.31083/j.rcm2305151.
