# Supplementary material for: Efficacy and Safety of Lomitapide in Homozygous Familial Hypercholesterolaemia: A Systematic Review
Source: Rev Cardiovasc Med. 2022 Apr 26;23(5):151. doi: 10.31083/j.rcm2305151 (PMC11273661; doi:10.31083/j.rcm2305151)
Supplement: Supplementary file 1 [file 2153-8174-23-5-151-s1.zip › 2153-8174-23-5-151-s1.docx]

Supplementary Table 1. PubMed search strategy.

| No. | Search items |
| --- | --- |
| #1 | (((((((((((((hypercholesterolemia [Title/Abstract]) OR (Hypercholesterolemias [Title/Abstract])) OR (High Cholesterol Levels [Title/Abstract])) OR (Cholesterol Level, High [Title/Abstract])) OR (Cholesterol Levels, High [Title/Abstract])) OR (High Cholesterol Level [Title/Abstract])) OR (Level, High Cholesterol [Title/Abstract])) OR (Levels, High Cholesterol [Title/Abstract])) OR (Elevated Cholesterol [Title/Abstract])) OR (Cholesterol, Elevated [Title/Abstract])) OR (Cholesterols, Elevated [Title/Abstract])) OR (Elevated Cholesterols [Title /Abstract])) OR (Hypercholesteremia [Title/Abstract])) OR (Hypercholesteremias [Title/Abstract]) |
| #2 | Search: (((((((lomitapide [Title/Abstract]) OR (BMS-201038 [Title/Abstract])) OR (BMS 201038 [Title/Abstract])) OR (lomitapide [Title/Abstract])) OR (Juxtapid [Title/Abstract])) OR (AEGR 733 [Title/Abstract])) OR (AEGR733 [Title/Abstract])) OR (AEGR-733 [Title/Abstract]) "lomitapide" [Title/Abstract] OR "BMS-201038" [Title/Abstract] OR "BMS-201038" [Title/Abstract] OR "lomitapide" [Title/ Abstract] OR "Juxtapid" [Title/Abstract] OR "AEGR-733" [Title/Abstract] OR "AEGR733" [Title/Abstract] OR "AEGR-733" [Title/Abstract] |
| #3 | #1 AND #2 |
